# Bacterial Viruses Subcommittee and Archaeal Viruses Subcommittee of the ICTV: update of taxonomy changes in 2021

**DOI:** 10.1007/s00705-021-05205-9

**Published:** 2021-08-21

**Authors:** Mart Krupovic, Dann Turner, Vera Morozova, Mike Dyall-Smith, Hanna M. Oksanen, Rob Edwards, Bas E. Dutilh, Susan M. Lehman, Alejandro Reyes, Diana P. Baquero, Matthew B. Sullivan, Jumpei Uchiyama, Jesca Nakavuma, Jakub Barylski, Mark J. Young, Shishen Du, Poliane Alfenas-Zerbini, Alla Kushkina, Andrew M. Kropinski, Ipek Kurtböke, J. Rodney Brister, Cédric Lood, B. L. Sarkar, Tong Yigang, Ying Liu, Li Huang, Johannes Wittmann, Nina Chanishvili, Leonardo J. van Zyl, Janis Rumnieks, Tomohiro Mochizuki, Matti Jalasvuori, Ramy K. Aziz, Małgorzata Łobocka, Kenneth M. Stedman, Andrey N. Shkoporov, Annika Gillis, Xu Peng, François Enault, Petar Knezevic, Rob Lavigne, Sung-Keun Rhee, Virginija Cvirkaite-Krupovic, Cristina Moraru, Andrea I. Moreno Switt, Minna M. Poranen, Andrew Millard, David Prangishvili, Evelien M. Adriaenssens

**Affiliations:** 1 Archaeal Virology Unit, Institut Pasteur, Paris, France; 2 Department of Applied Sciences, Faculty of Health and Applied Sciences, University of the West of England, Bristol, UK; 3 Laboratory of Molecular Microbiology, Institute of Chemical Biology and Fundamental Medicine SB RAS, Novosibirsk, Russia; 4 Veterinary Biosciences, Faculty of Veterinary and Agricultural Sciences, University of Melbourne, Parkville, Australia; 5 Molecular and Integrative Biosciences Research Programme, Faculty of Biological and Environmental Sciences, University of Helsinki, Helsinki, Finland; 6 Flinders Accelerator for Microbiome Exploration, Adelaide, Australia; 7 Theoretical Biology and Bioinformatics, Department of Biology, Utrecht University, Utrecht, Netherlands; 8 Center for Biologics Evaluation and Research, US Food and Drug Administration, 10903 New Hampshire Ave, Silver Spring, MD 20903 USA; 9 Max Planck Tandem Group in Computational Biology, Department of Biological Sciences, Universidad de Los Andes, Bogotá, Colombia; 10 Department of Microbiology, The Ohio State University, Columbus, OH 43210 USA; 11 Department Civil, Environmental, and Geodetic Engineering, The Ohio State University, Columbus, OH 43210 USA; 12 School of Veterinary Medicine, Azabu University, Sagamihara, Japan; 13 College of Veterinary Medicine, Animal Resources and Biosecurity, Makerere University, Kampala, Uganda; 14 Department of Molecular Virology, Faculty of Biology, Institute of Experimental Biology, Adam Mickiewicz University in Poznan, Collegium Biologicum-Umultowska 89, 61-614 Poznan, Poland; 15 Department of Plant Sciences and Plant Pathology, Montana State University, Bozeman, MT USA; 16 Department of Microbiology, College of Life Sciences, Wuhan University, Wuhan, China; 17 Department of Microbiology, Universidade Federal de Viçosa, Viçosa, Brazil; 18 Zabolotny Institute of Microbiology and Virology, NAS of Ukraine, Kyiv, Ukraine; 19 Department of Food Science, University of Guelph, Guelph, Canada; 20 Department of Pathobiology, University of Guelph, Guelph, Canada; 21 University of the Sunshine Coast, Sippy Downs, Australia; 22 National Center for Biotechnology Information, National Library of Medicine, National Institutes of Health, Bethesda, USA; 23 KU Leuven, Leuven, Belgium; 24 Emeritus ICMR-National Institute of Cholera and Enteric Diseases (NICED), Kolkata, India; 25 Beijing University of Chemical Technology, Beijing, China; 26 State Key Laboratory of Microbial Resources, Institute of Microbiology, Chinese Academy of Sciences, Beijing, China; 27 Leibniz Institute DSMZ-German Collection of Microorganisms and Cell Cultures GmbH, Berlin, Germany; 28 The Eliava Institute of Bacteriophage, MIcrobiology and Virology, Tbilisi, Georgia; 29 University of the Western Cape, Western Cape, South Africa; 30 Latvian Biomedical Research and Study Center, Riga, Latvia; 31 Earth-Life Science Institute, Tokyo Institute of Technology, Tokyo, Japan; 32 University of Jyvaskyla, Jyvaskyla, Finland; 33 Department of Microbiology and Immunology, Faculty of Pharmacy, Cairo University and Microbiology and Immunology Research Program, Children’s Cancer Hospital Egypt, Cairo, 57357 Egypt; 34 Laboratory of Bacteriophage Biology, Institute of Biochemistry and Biophysics of the Polish Academy of Sciences, Warsaw, Poland; 35 Biology Department and Center for Life in Extreme Environments, Portland State University, Portland, USA; 36 University College Cork, Cork, Ireland; 37 Laboratory of Food and Environmental Microbiology, Earth and Life Institute, UC Louvain, Louvain-la-Neuve, Belgium; 38 Microbial Immunity Group, Department of Biology, University of Copenhagen, Copenhagen, Denmark; 39 Université Clermont Auvergne, CNRS, LMGE, 63000 Clermont-Ferrand, France; 40 Faculty of Sciences Department of Biology and Ecology, University of Novi Sad, Novi Sad, Serbia; 41 Department of Biological Sciences and Biotechnology, Chungbuk National University, Cheongju, South Korea; 42 Institute for Chemistry and Biology of the Marine Environment, University of Oldenburg, Oldenburg, Germany; 43 Escuela de Medicina Veterinaria, Pontificia Universidad Católica de Chile, Santiago, Chile; 44 Department of Genetics and Genome Biology, University of Leicester, Leicester, UK; 45 Ivane Javakhishvili Tbilisi State University, Tbilisi, Georgia; 46 Quadram Institute Biosciences, Norwich Research Park, Norwich, NR4 7UQ UK

## Abstract

In this article, we – the Bacterial Viruses Subcommittee and the Archaeal Viruses Subcommittee of the International Committee on Taxonomy of Viruses (ICTV) – summarise the results of our activities for the period March 2020 – March 2021. We report the division of the former Bacterial and Archaeal Viruses Subcommittee in two separate Subcommittees, welcome new members, a new Subcommittee Chair and Vice Chair, and give an overview of the new taxa that were proposed in 2020, approved by the Executive Committee and ratified by vote in 2021. In particular, a new realm, three orders, 15 families, 31 subfamilies, 734 genera and 1845 species were newly created or redefined (moved/promoted).

**Supplementary Information:**

The online version contains supplementary material available at 10.1007/s00705-021-05205-9.

## Changes in Subcommittee structure and membership

Bacteriophage and archaeal virus taxonomy has been formally under the auspices of the ICTV Bacterial and Archaeal Viruses Subcommittee, which, at its inception in 1966, was named the Viruses of Prokaryotes Subcommittee, led by David E. Bradley (https://talk.ictvonline.org/information/w/ictv-history). Given the revived interest in bacterial and archaeal viruses and recent enormous increase in the number of characterized isolates and need for creation of numerous taxa to classify them, the Executive Committee (EC) voted on the creation of two separate Subcommittees (EC51, July 2019), formally starting their mandates after EC52 (October 2020). The new Bacterial Viruses Subcommittee is chaired by Evelien Adriaenssens, supported by Dann Turner as the Vice Chair, and the new Archaeal Viruses Subcommittee is chaired by Mart Krupovic. Both Chairs were elected for three-year terms ending in 2023. As a result, this taxonomy update summarises both bacterial and archaeal virus proposals for the last time, reflecting the fact that proposals were submitted prior to the formal reorganisation of the original Subcommittee.

In the new Bacterial Viruses Subcommittee, we continue our structure of Study Groups (SGs), regional representatives and general members. We would like to welcome new members Jesca Nakavuma (Uganda), Alejandro Reyes (Colombia), Cristina Moraru (Germany), Susan Lehman (USA), Cédric Lood (Belgium) and Andrey Shkoporov (Ireland) and would like to thank those who have left the Subcommittee for their service.

In the framework of the Bacterial and Archaeal Viruses Subcommittee, all taxonomic proposals dealing with archaeal viruses were handled by a single SG. The new Archaeal Viruses Subcommittee currently includes 11 SGs (Table 1), each created for a three-year term. Of note, the *Halopanivirales* SG, chaired by Hanna M. Oksanen (Finland), oversees the taxonomy of evolutionarily related viruses of the order *Halopanivirales*, which includes two families of viruses infecting archaea (*Sphaerolipoviridae* and *Simuloviridae*) and one family of bacteriophages (*Matsushitaviridae*). Thus, the latter SG bridges the two Subcommittees. Additional SGs will be created in the near future, in particular, to develop the taxonomy of archaeal members of the class *Caudoviricetes*; only a handful of isolated representatives of this highly diverse and expansive group of archaeal viruses are currently classified.

**Table 1:** Composition of the archaeal viruses subcommittee

Study Group	Member	Country
*Bicaudaviridae* SG	Mart Krupovic*	France
	Li Huang	China
	Mark J. Young	USA
	Virginija Cvirkaite-Krupovic	France
Desulfurococcales viruses SG	Tomohiro Mochizuki*	Japan
	Mart Krupovic	France
*Fuselloviridae* SG	Kenneth M. Stedman*	USA
	Mart Krupovic	France
*Halopanivirales* SG	Hanna M. Oksanen*	Finland
	Mike Dyall-Smith	Australia
	Shishen Du	China
	Matti Jalasvuori	Finland
*Halspiviridae* SG	Mike Dyall-Smith*	Australia
	Hanna M. Oksanen	Finland
*Ovaliviridae* SG	Li Huang*	China
*Pleolipoviridae* SG	Hanna M. Oksanen*	Finland
	Mike Dyall-Smith	Australia
	Ying Liu	France
*Portogloboviridae* SG	Mart Krupovic*	France
	Ying Liu	France
*Thaspiviridae* SG	Sung-Keun Rhee*	Republic of Korea
	Mart Krupovic	France
*Tokiviricetes* SG	Mart Krupovic*	France
	Tomohiro Mochizuki	Japan
	Xu Peng	Denmark
	Diana P. Baquero	France
*Turriviridae* SG	Mark J. Young*	USA

*Study Group Chair

## Taxonomy update

We had a record year of submissions of taxonomy proposals in 2020, with 188 proposals submitted, of which all but one were approved and ratified (Supplementary Table S1). The changes at each of the taxonomic ranks in use for bacterial and archaeal viruses are summarised in Table 2. We created a record 1775 new species, 700 new genera, 28 new subfamilies, 14 new families and two new orders and also created one new realm containing one new kingdom, one new phylum and one new class. Given the large numbers of proposals and taxa that were created, moved or renamed, it becomes unfeasible to describe all the changes in detail; however, we urge interested readers to consult Supplementary Table S1 and the associated proposals from the ICTV website (https://talk.ictvonline.org/files/ictv_official_taxonomy_updates_since_the_8th_report/m/prokaryote-official). Instead, below we provide a brief overview of the most notable changes.

**Table 2:** Summary of taxonomic changes for bacterial and archaeal viruses for Master Species List 36, ratified March 2021

	Species	Genus	Subfamily	Family	Order	Class	Phylum	Kingdom	Realm
Abolished	20	2	0	0	0	0	0	0	0
New	1775	700	28	14	2	1	1	1	1
Moved or promoted (and/or renamed)	70	34	3	1	1	0	0	0	0
Renamed	33	8	0	1	1	1	0	0	0

## The new realm *Adnaviria*, a new megataxonomy of archaeal filamentous viruses

Recently, the taxonomic framework of the ICTV has been expanded from five to 15 ranks, with the highest-level rank, realm, being equivalent to the domain rank used for cellular organisms [1]. Until recently, four such realms had been established for classification of viruses infecting hosts from different domains of life [2, 3]. This year, a new realm, *Adnaviria*, was created for classification of archaeal filamentous viruses with dsDNA genomes that adopt the A-form conformation within their virions [4, 5]. The realm includes three families, *Tristromaviridae*, *Rudiviridae* and *Lipothrixviridae*, which are evolutionarily related to each other, but not to any other known group of viruses. The families *Rudiviridae* and *Lipothrixviridae* were already grouped together in the order *Ligamenvirales* [6], and a single-family order, *Primavirales*, has now been created for the family *Tristromaviridae*. The two orders are unified in the class *Tokiviricetes*, kingdom *Zilligvirae* and realm *Adnaviria*.

## Class *Caudoviricetes*, order *Caudovirales*

We delineated three new families of short-tailed phages with small genomes that were formerly assigned to the family *Podoviridae*. The family *Salasmaviridae*, named in honour of Margarita Salas Falgueras, comprises the existing subfamily *Picovirinae*, which includes the classical bacillus phage φ29 and a range of related *Bacillus*-infecting phages with genomes between 18 and 27 kb in size. The family *Rountreeviridae*, named in honour of Phyllis Margaret Rountree, groups *Enterococcus*-infecting phages with genomes between 17 and 19 kb in size, whereas the family *Guelinviridae*, named after Antonina Guelin, groups *Clostridium*-infecting phages with genome sizes between 16 and 19 kb.

The new family *Schitoviridae*, named after Giancarlo Schito, is the formalisation of the group of N4-like phages defined by the presence of a large virion-associated RNA polymerase, described in more detail in reference [7].

The new family *Zobellviridae*, named after Claude Zobell, groups a set of globally distributed podoviruses associated with marine ecosystems first proposed by Bischoff and colleagues [8].

## Class *Leviviricetes*

Based on the investigation by Callanan and colleagues on the expansion of known ssRNA virus genomes [9], the family *Leviviridae* was elevated to the rank of class, named *Leviviricetes* (replacing the class *Allassoviricetes*), and expanded to include two orders (*Norzivirales* and *Timlovirales*) and six new families: *Fiersviridae* (renamed from the original family *Leviviridae*), *Atkinsviridae*, *Duinviridae*, *Solspiviridae*, *Blumeviridae* and *Steitzviridae*. A detailed description of the new taxa will be published separately.

## Class *Tectiliviricetes*, new family *Autolykiviridae*

The new family *Autolykiviridae* formalises the group of non-tailed dsDNA bacteriophages discovered and described by Kauffmann and colleagues [10], combining features of both corticoviruses and tectiviruses. This new family contains two new genera and five new species.

## Order *Halopanivirales*, new families *Matsushitaviridae* and *Simuloviridae*

Until recently, the family *Sphaerolipoviridae*, which includes icosahedral tailless viruses with internal membranes, consisted of three genera, *Alphasphaerolipovirus*, *Betasphaerolipovirus* and *Gammasphaerolipovirus*. The first two of these genera included viruses infecting halophilic archaea, whereas the last one included phages infecting thermophilic bacteria [11]. Although viruses from the three genera are evolutionarily related [12], they display considerable sequence divergence. Thus, the genera *Betasphaerolipovirus* and *Gammasphaerolipovirus* have been renamed and moved from the *Sphaerolipoviridae* into new families, *Simuloviridae* and *Matsushitaviridae* (named after Isao Matsushita), respectively. The order *Halopanivirales* now contains a family of bacterial viruses, *Matsushitaviridae,* and two families of archaeal viruses, family *Sphaerolipoviridae* and *Simuloviridae*. As mentioned above, the order is under purview of a single Study Group, which is part of both the Archaeal Viruses SC and the Bacterial Viruses SC.

## Order *Tubulavirales*, new family *Paulinoviridae*

The new family *Paulinoviridae* addresses the challenge of defining family demarcation criteria for phages with small genomes, where members of the same family would share at least two orthologous proteins. Informed by prior work on a gene-content network of predicted filamentous prophage sequences [13], we moved the genera *Bifilivirus* and *Thomixvirus* from the family *Inoviridae* to the family *Paulinoviridae*.

## Online (10th) Report of the ICTV

Virus Taxonomy: The Classification and Nomenclature of Viruses - The Online (10th) Report of the ICTV is freely accessible at http://ictv.global/report, and summaries of the chapters on each virus family are published in the Journal of General Virology. In 2020, four new chapters on bacterial and archaeal viruses were produced by members of the Archaeal Viruses SC and Bacterial Viruses SC, namely, on the families *Herelleviridae* [14], *Spiraviridae* [15], *Ovaliviridae* [16] and *Finnlakeviridae* [17].

## Conclusion

This past year has been extremely productive in terms of new bacterial and archaeal virus taxa described. It would not have been possible without an active pool of scientists, both within and outside the subcommittee and its study groups, who scour databases, perform analyses and submit proposals. We continue to encourage people to contact us to formalise new discoveries into the taxonomic framework and will keep reaching out to interested parties. Finally, we would like to acknowledge one person in particular, Prof Andrew Kropinski, the Subcommittee Chair from 2014-2020, who authored, co-authored and/or assisted with the majority of proposals that have been approved in the last decade.

## Supplementary Information

Below is the link to the electronic supplementary material.

Supplementary file1 (PDF 175 KB)
