# Supplementary material for: Bacterial Viruses Subcommittee and Archaeal Viruses Subcommittee of the ICTV: update of taxonomy changes in 2021
Source: Arch Virol. 2021 Aug 21;166(11):3239–44. doi: 10.1007/s00705-021-05205-9 (PMC8497307; doi:10.1007/s00705-021-05205-9)
Supplement: Supplementary file 1 — Supplementary file1 (PDF 175 KB) [file 705_2021_5205_MOESM1_ESM.pdf]

SUPPLEMENTARY TABLE 1: OVERVIEW OF ALL RATIFIED PROPOSALS, SUBMITTED 2020, APPROVED BY THE EC BY EMAIL VOTE NOVEMBER 2020, RATIFIED BY THE ICTV MEMBERSHIP MARCH 2021.

Authors of the Taxonomy Proposal	Taxonomic change	Code
**Archaeal Viruses Proposals**		
Dyall-Smith ML, Witte A, Oesterhelt D, Pfeiffer F	Create two new species in the genus *Myohalovirus* (*Caudovirales: Myoviridae*)	2020.001B.
Dyall-Smith ML, Tang S-L	Create one new genus (*Haloferacalesvirus*) including five new species (*Caudovirales*: *Myoviridae*)	2020.002B.
Krupovic M	Create two new species in the *genus Bottigliavirus* (*Ampullaviridae*)	2020.009B.
Krupovic M, Baquero DP, Prangishvili D	Create six new genera and 14 new species (*Ligamenvirales*: *Rudiviridae*)	2020.141B.
Krupovic M, Baquero DP, Prangishvili D	Rename the genus *Alphaglobulovirus* and create two new species (*Globuloviridae*)	2020.063B.
Liu Y, Du S, Chen X, Krupovic M	Create one new family (*Simuloviridae*) including one genus (*Yingchengvirus* - formerly *Betasphaerolipovirus*) moved from the family *Sphaerolipoviridae* (*Halopanivirales*)	2020.151B.
Krupovic M, Baquero DP, Prangishvili D	Create one new genus (*Betatristromavirus*) including one existing species (*Thermoproteus tenax virus 1*) and create one new species in the genus *Alphatristromavirus* (*Tristromaviridae*)	2020.168B.
Krupovic M, Kuhn JH, Wang F, Baquero DP, Egelman EH, Koonin EV, Prangishvili D	Create one new realm (*Adnaviria*) for classification of filamentous archaeal viruses with linear dsDNA genomes	2020.186B.
**Bacterial Viruses Proposals**		
Lehman SM, Adriaenssens EM	Abolish the species Pseudomonas virus 42 (*Caudovirales*: *Myoviridae*)	2020.003B.
Adriaenssens EM, Tolstoy I, Moraru C, Kropinski AM, Turner D	Abolish the genus *Viunavirus*, move six existing species to the genus *Kuttervirus* and create 30 new species (*Caudovirales*: *Ackermannviridae*)	2020.004B.
Maina AN, Kropinski AM, Tolstoy I, Adriaenssens EM, Moraru C, Turner D	Create six new genera (*Caudovirales*: *Ackermannviridae*)	2020.005B.
Adriaenssens EM, Tolstoy I, Kropinski AM, Moraru C	Correct minor errors in the taxonomy of four actinobacteriophage-related genera (*Caudovirales*: *Siphoviridae*)	2020.006B.
Adriaenssens EM, Tolstoy I, Moineau S, Kropinski AM	Create one new genus (*Agmunavirus*) including one species (*Caudovirales*: *Siphoviridae*)	2020.007B.
Adriaenssens EM, Tolstoy I, Turner D, Kropinski AM	Create one new genus (*Alachuavirus*) including one species (*Caudovirales*: *Siphoviridae*)	2020.008B.

Adriaenssens EM, Tolstoy I, Łobocka M, Moraru C, Barylski J, Tong Y, Kropinski AM, Turner D	Create one new genus (*Anjalivirus*) including two species (*Caudovirales*: *Podoviridae*)	2020.010B.
Adriaenssens EM, Tolstoy I, Turner D, Kropinski AM	Create one new genus (*Annadreamyvirus*) including two species (*Caudovirales*: *Siphoviridae*)	2020.011B.
Adriaenssens EM, Turner D, Tolstoy I, Kropinski AM	Create one new genus (*Arawnvirus*) including one species (*Caudovirales*: *Siphoviridae*)	2020.012B.
Lehman SM, Petit MA, Lossouarn J, Tong Y, Tolstoy I, Adriaenssens EM	Create ten new genera (*Caudovirales*: *Siphoviridae*)	2020.013B.
Adriaenssens EM, Tolstoy I, Łobocka M, Moraru C, Barylski J, Tong Y, Kropinski AM	Create one new genus (*Astrithrvirus*) including one species (*Caudovirales*: *Podoviridae*)	2020.014B.
Adriaenssens EM, Tolstoy I, Turner D, Lueder M, Mahony J, Neve H, Moineau S, Kropinski AM	Create one new genus (*Audreyjarvisvirus*) including seven species (*Caudovirales*: *Siphoviridae*)	2020.015B.
Kauffman KM, Hussain FA, Yang J, Arevalo P, Brown JM, Chang WK, Van Insberghe D, Elsherbini J, Sharma RS, Cutler MB, Kelly L, Polz MF	Create one new family (*Autolykiviridae*) of non-tailed dsDNA bacterial viruses in the double jelly roll fold major capsid lineage	2020.016B.
Adriaenssens EM, Tolstoy I, Moraru C, Kropinski AM	Create one new genus (*Ayohtrevirus*) including one species (*Caudovirales*: *Myoviridae*)	2020.017B.
Adriaenssens EM, Tolstoy I, Kropinski AM, Moraru C, Łobocka M	Create one new subfamily (*Azeredovirinae*) including one new genus (*Dubowvirus*) (*Caudovirales*: *Siphoviridae*)	2020.018B.
Adriaenssens EM, Tolstoy I, Łobocka M, Moraru C, Barylski J, Tong Y, Kropinski AM	Create one new genus (*Badaztecvirus*) including two species (*Caudovirales*: *Podoviridae*)	2020.019B.
Adriaenssens EM, Turner D, Tolstoy I, Kropinski AM	Split the genus *Barnyardvirus* into three genera, two of which (*Konstantinevirus*, *Predatorvirus*) are new (*Caudovirales*: *Siphoviridae*)	2020.020B.
Kropinski AM, Tolstoy I, Adriaenssens EM, Turner D, Barylski J	Create one new genus (*Beceayunavirus*) including one new species (*Caudovirales*: *Siphoviridae*)	2020.021B.
Adriaenssens EM, Tolstoy I, Kropinski AM Barylski J	Create one new genus (*Becedseptimavirus*) including one new species (*Caudovirales*: *Myoviridae*)	2020.022B.
Adriaenssens EM, Tolstoy I, Turner D, Kropinski AM	Create one new subfamily (*Beephvirinae*) including three new genera (*Caudovirales*: *Podoviridae*)	2020.023B.
Kropinski AM, Tolstoy I, Turner D, Adriaenssens EM	Create one new genus (*Borockvirus*) including one new species (*Caudovirales*: *Myoviridae*)	2020.024B.
Adriaenssens EM, Tolstoy I, Kropinski AM, Moraru C, Łobocka M	Create one new subfamily (*Bronfenbrennervirinae*) including the new genus Peeveelvirus (*Caudovirales*: *Siphoviridae*)	2020.025B.
Adriaenssens EM, Tolstoy I, Turner D, Kropinski AM	Create one new genus (*Burrovirus*) including three new species (*Caudovirales*: *Podoviridae*)	2020.026B.

Adriaenssens EM, Tolstoy I, Kropinski AM, Barylski J	Create one new genus (*Camtrevirus*) including three new species (*Caudovirales*: *Siphoviridae*)	2020.027B.
Adriaenssens EM, Tolstoy I, Kropinski AM, Wittmann J, Buttimer C, Coffey A	Create one new genus (*Cbunavirus*) including four new species (*Caudovirales*: *Schitoviridae*)	2020.028B.
Adriaenssens EM, Tolstoy I, Moraru C, Turner D, Lueder M, Mahony J, Neve H, Moineau S, Kropinski AM	Create 34 new species in the genus *Ceduovirus* (*Caudovirales*: *Siphoviridae*)	2020.029B.
Anany H, Moraru C, Turner D, Adriaenssens EM, Kropinski AM	Create two new subfamilies and three new genera (*Caudovirales*: *Chaseviridae*)	2020.030B.
Adriaenssens EM, Tolstoy I, Turner, D Kropinski AM, Moraru C	Reassessment of the genus *Cheoctovirus* and formation of one new genus *Avanivirus* (*Caudovirales*: *Siphoviridae*)	2020.031B.
Adriaenssens EM, Tolstoy I, Turner D, Lueder M, Moineau S, Kropinski AM	Create one new genus (*Chertseyvirus*) including one new species (*Caudovirales*: *Siphoviridae*)	2020.032B.
Adriaenssens EM, Tolstoy I, Kropinski AM, Moraru C	Create nine new species in the genus *Chivirus* (*Caudovirales*: *Siphoviridae*)	2020.033B.
Kropinski AM, Tolstoy I, Adriaenssens EM, Moineau S, Mahony J, Lueder M, Neve H	Create one new genus (*Chopinvirus*) including one new species (*Caudovirales*: *Podoviridae*)	2020.034B.
Adriaenssens EM, Tolstoy I, Turner D, Kropinski AM	Taxonomic analysis of the myoviruses infecting *Clostridioides difficile* (*Caudovirales*: *Myoviridae*)	2020.035B.
Adriaenssens EM, Tolstoy I, Turner D, Kropinski AM	Create one new genus of *Mycobacterium* siphoviruses (*Cornievirus*) including one new species (*Caudovirales*: *Siphoviridae*)	2020.036B.
Adriaenssens EM, Tolstoy I, Turner D, Kropinski AM	Correction of errors in the master species list (Caudovirales)	2020.037B.
Adriaenssens EM, Tolstoy I, Kropinski AM, Łobocka M	Create one new genus (*Coventryvirus*) including seven new species (*Caudovirales*: *Siphoviridae*)	2020.038B.
Adriaenssens EM, Tolstoy I, Turner D, Kropinski AM	Create three new genera (*Cukevirus*, *Indlulamithivirus* and *Fowlmouthvirus*) each with one new species (*Caudovirales*: *Siphoviridae*)	2020.040B.
Adriaenssens EM, Tolstoy I, Turner D, Kropinski AM	Create one new subfamily (*Deejayvirinae*) including three new genera (*Caudovirales*: *Siphoviridae*)	2020.041B.
Caruso SM, deCarvalho TN, Erill I, Gill J, Gillis A	Create two new genera (*Deltatectivirus* and *Epsilontectivirus*) including three new species (*Kalamavirales*: *Tectiviridae*)	2020.042B.
Adriaenssens EM, Tolstoy I, Turner D, Kropinski AM	Create 29 new species in two existing genera (*Caudovirales*: *Demerecviridae*)	2020.043B.
Wittmann J, Adriaenssens EM, Kropinski AM	Create one new genus (*Dendoorenvirus*) including one new species (*Caudovirales*: *Schitoviridae*)	2020.044B.
Adriaenssens EM, Tolstoy I, Kropinski AM, Barylski J, Gillis A	Create one new genus (*Deurplevirus*) including one new species (*Caudovirales*: *Siphoviridae*)	2020.045B.

Sadunishvili T, Kvesitadze G, Kropinski AM, Adriaenssens EM, Truncaitė L, Šimoliūnas E	Create one new genus (*Dibbivirus*) including three new species (*Caudovirales*: *Myoviridae*)	2020.046B.
Kropinski AM, Adriaenssens EM, Barylski J	Create one new genus (*Donellivirus*) to include the species *Bacillus virus G* (*Caudovirales*: *Myoviridae*)	2020.047B.
Adriaenssens EM, Tolstoy I, Moraru C Kropinski AM	Create five new genera (*Caudovirales*: *Drexlerviridae*)	2020.048B.
Adriaenssens EM, Tolstoy I, Turner D, Kropinski AM	Create one new species in the genus *Ruthyvirus* (*Caudovirales*: *Siphoviridae*)	2020.049B.
Wittmann J, Adriaenssens EM, Kropinski AM	Create one new genus (*Eceepunavirus*) including one new species (*Caudovirales*: *Schitoviridae*)	2020.050B.
Adriaenssens EM, Tolstoy I, Turner D, Kropinski AM	Create one new subfamily (*Eekayvirinae*) including two new genera (*Caudovirales*: *Podoviridae*)	2020.051B.
Kropinski AM, Tolstoy I, Adriaenssens EM, Barylski J	Create one new genus (*Elmenteitavirus*) including one new species (*Caudovirales*: *Myoviridae*)	2020.052B.
Kropinski AM, Tolstoy I, Turner D, Adriaenssens EM	Create one new subfamily (*Emmerichvirinae*) including two new genera (*Ishigurovirus* and *Ceceduovirus*) (*Caudovirales*: *Myoviridae*)	2020.053B.
Adriaenssens EM, Tolstoy I, Kropinski AM, Moraru C, Wittmann J	Create one subfamily (*Enquatrovirinae*) including three new genera (*Caudovirales*: *Schitoviridae*)	2020.054B.
Kirchberger PC, Ochman H	Create one new genus (*Enterogokushovirus*) in the subfamily *Gokushovirinae* (*Microviridae*)	2020.055B.
Adriaenssens EM, Tolstoy I, Kropinski AM, Moraru C, Wittmann J	Create one new subfamily (*Erskinevirinae*) including two genera (*Caudovirales*: *Podoviridae*)	2020.056B.
Adriaenssens EM, Tolstoy I, Moraru C, Kropinski AM, Tsourkas PK, Barylski J	Create 12 new species in the genus *Fernvirus* (formerly *Sitaravirus*) (*Caudovirales*: *Siphoviridae*)	2020.057B.
Nilsson E, Holmfeldt K	Create six new genera including five new species in the genus *Muminvirus*, six new species in the genus *Lillamyvirus*, one new species in the genus *Hattifnattvirus*, two new species in the genus *Pippivirus*, one new species in the genus *Tantvirus* and one new species in the genus *Labanvirus* (*Caudovirales*)	2020.058B.
Adriaenssens EM, Tolstoy I, Moraru C, Turner D, Lueder M, Mahony J, Neve H, Moineau S, Kropinski AM	Create one new genus (*Fremauxvirus*) including two new species (*Caudovirales*: *Siphoviridae*)	2020.059B.
Adriaenssens EM, Tolstoy I, Kropinski AM, Jang HB, Sullivan MB, Moraru C, Wittmann J	Create one new subfamily (*Fuhrmanvirinae*) including two new genera (*Caudovirales*: *Schitoviridae*)	2020.060B.
Kropinski AM, Turner D, Adriaenssens EM	Create one new genus (*Fukuivirus*) including one new species and one existing species (*Microcystis virus Ma-LMM01*) (*Caudovirales*: *Myoviridae*)	2020.061B.
Adriaenssens EM, Tolstoy I, Turner D, Kropinski AM	Create one new genus (*Gilsonvirus*) including two new species (*Caudovirales*: *Siphoviridae*)	2020.062B.

Adriaenssens EM, Tolstoy I, Kropinski AM, Tsourkas PK	Create one new subfamily (*Gochnauervirinae*) including four genera (*Caudovirales*: *Siphoviridae*)	2020.064B.
Adriaenssens EM, Tolstoy I, Kropinski AM, Moraru C	Create one new subfamily (*Gorgonvirinae*) including two genera (*Caudovirales*: *Myoviridae*)	2020.065B.
Adriaenssens EM, Tolstoy I, Łobocka M, Moraru C, Barylski J, Tong Y, Kropinski AM	Create one new family (*Guelinviridae*) of *Clostridium* phages including one new subfamily, four new genera and nine new species (*Caudovirales*)	2020.066B.
Adriaenssens EM, Tolstoy I, Kropinski AM, Barylski J	Create one new subfamily (*Gutmannvirinae*) including two new genera and three new species (*Caudovirales*: *Siphoviridae*)	2020.067B.
Adriaenssens EM, Tolstoy I, Moraru C, Kropinski AM, Tsourkas PK, Barylski J	Rename the genus *Halcyonevirus* (formerly *Trippvirus*) and create four new species (*Caudovirales*: *Siphoviridae*)	2020.068B.
Adriaenssens EM, Tolstoy I, Moraru C, Kropinski AM	Create 13 new species in the genus *Hanrivervirus* (*Caudovirales*: *Drexlerviridae*)	2020.069B.
Lehman SM, Petit MA, Lossouarn J, Tong Y, Tolstoy I, Adriaenssens E	Promote the genus *Hendrixvirus* to the rank of subfamily (*Hendrixvirinae*) and establish nine new genera (*Caudovirales*: *Siphoviridae*)	2020.070B.
Adriaenssens EM, Tolstoy I, Turner D, Kropinski AM	Create one new genus (*Henunavirus*) including two new species in the subfamily *Vequintavirinae* (*Caudovirales*: *Myoviridae*)	2020.071B.
Adriaenssens EM, Tolstoy I, Kropinski AM, Moraru C, Hertel R, Lehman SM, Barylski J	Create 13 new genera and move two genera (*Caudovirales*: *Herelleviridae*)	2020.072B.
Adriaenssens EM, Tolstoy I, Turner D, Kropinski AM	Create one new genus (*Hnatkovirus*) including one new species (*Caudovirales*: *Siphoviridae*)	2020.073B.
Adriaenssens EM, Tolstoy I, Kropinski AM, Barylski J	Create one new genus (*Hubeivirus*) including two new species (*Caudovirales*: *Siphoviridae*)	2020.074B.
Adriaenssens EM, Tolstoy I, Kropinski AM, Moraru C, Wittmann J	Create one new genus (*Huelvavirus*) including one new species (*Caudovirales*: *Schitoviridae*)	2020.075B.
Adriaenssens EM, Tolstoy I, Kropinski AM, Moraru C, Wittmann J	Create one new subfamily (*Humphriesvirinae*) including three existing genera (*Caudovirales*: *Schitoviridae*)	2020.076B.
Adriaenssens EM, Tolstoy I, Moraru C Kropinski AM	Create three new species in the genus *Ilzatvirus* (*Caudovirales*: *Siphoviridae*)	2020.077B.
Adriaenssens EM, Tolstoy I, Kropinski AM, Barylski J	Create one new genus (*Jarrellvirus*) including one new species (*Caudovirales*: *Siphoviridae*)	2020.078B.
Adriaenssens EM,Tolstoy I, Kropinski AM, Morozova V, Barylski J	Create one new genus (*Kamchatkavirus*) including one new species (*Caudovirales*: *Siphoviridae*)	2020.079B.
Kropinski AM, Adriaenssens EM, Turner D	Create one new genus (*Kanagawavirus*) including two new species (*Caudovirales*: *Myoviridae*)	2020.080B.
Adriaenssens EM, Tolstoy I, Turner D, Kropinski AM	Create one new genus (*Karimacvirus*) including five new species (*Caudovirales*: *Siphoviridae*)	2020.081B.

Kropinski AM, Adriaenssens EM, Neve H, Franz CMAP, Sprotte S	Create one new genus (*Knuthellervirus*) including one new species (*Caudovirales*: *Siphoviridae*)	2020.082B.
Kropinski AM, Turner D, Adriaenssens EM	Create one new genus (*Kozyakovvirus*) including one new species (*Caudovirales*: *Podoviridae*)	2020.083B.
Adriaenssens EM, Tolstoy I, Moraru C, Kropinski AM	Create one new genus (*Kuleanavirus*) including one new species (*Caudovirales*: *Siphoviridae*)	2020.084B.
Adriaenssens EM, Tolstoy I, Kropinski AM	Create one new genus (*Kungbxnavirus*) including one new species (*Caudovirales*: *Myoviridae*)	2020.085B.
Adriaenssens EM, Tolstoy I, Turner D, Kropinski AM	Create one new genus (*Kylevirus*) including one new species (*Caudovirales*: *Myoviridae*)	2020.086B.
Kabwe M, Petrovski S, Tucci J, Adriaenssens EM, Tolstoy I, Kropinski AM	Create one new genus (*Lahexavirus*) including four new species (*Caudovirales*: *Podoviridae*)	2020.087B.
Adriaenssens EM, Tolstoy I, Turner D, Kropinski AM	Create one new genus (*Lambovirus*) including five new species (*Caudovirales*: *Siphoviridae*)	2020.088B.
Kropinski AM, Tolstoy I, Turner D, Adriaenssens EM	Create 12 new genera including 17 new species for *Lactobacillus* viruses (*Caudovirales*: *Siphoviridae*)	2020.089B.
Adriaenssens EM, Tolstoy I, Moraru C, Kropinski AM	Create three new species in the genus *Laroyevirus* (*Caudovirales*: *Siphoviridae*)	2020.090B.
Adriaenssens EM, Tolstoy I, Kropinski AM	Create one new genus (*Lastavirus*) including two new species (*Caudovirales*: *Podoviridae*)	2020.091B.
Kropinski AM, Tolstoy I, Adriaenssens EM, Kabwe M, Tucci J	Create one new genus (*Latrobevirus*) including one new species (*Caudovirales*: *Siphoviridae*)	2020.092B.
Adriaenssens EM, Tolstoy I, Turner D, Kropinski AM	Create one new genus (*Leicestervirus*) including three new species (*Caudovirales*: *Siphoviridae*)	2020.093B.
Adriaenssens EM, Tolstoy I, Moraru C Kropinski AM	Create one new genus (*Seongbukvirus*) including one new species and create four new species of *Leuconostoc* phages in two existing genera (*Caudovirales*: *Siphoviridae*)	2020.094B.
Callanan J, Stockdale SR, Adriaenssens EM, Kuhn JH, Rumnieks J, Shkoporov A, Draper LA, Ross RP, Hill C	Rename one class (*Leviviricetes* - formerly *Allassoviricetes*), rename one order (*Norzivirales* - formerly *Levivirales*), create one new order (*Timlovirales*), and expand the class to a total of six families, 420 genera and 883 species	2020.095B.
Truncaitė L, Kropinski AM, Adriaenssens EM, Šimoliūnas E	Create one new genus (*Lietduovirus*) including one new species (*Caudovirales*: *Myoviridae*)	2020.096B.
Wittmann J, Adriaenssens EM, Kropinski AM	Create one new genus (*Littlefixvirus*) containing one new species (*Caudovirales*: *Schitoviridae*)	2020.097B.
Kropinski AM, Tolstoy I, Turner D, Adriaenssens EM	Create one new genus (*Marfavirus*) including two new species (*Caudovirales*: *Myoviridae*)	2020.098B.
Adriaenssens EM, Tolstoy I, Moraru C, Kropinski AM	Create one new species in the genus *Marthavirus* (*Caudovirales*: *Myoviridae*)	2020.099B.
Oksanen HM, Krupovic M, Jalasvuori M	Create one new family (*Matsushitaviridae*) including one renamed genus (*Hukuchivirus* – formerly	2020.100B.

	Gammasphaerolipovirus) moved from the family *Sphaerolipoviridae* (*Halopanivirales*)	
Adriaenssens EM, Tolstoy I, Kropinski AM, Ramsey J	Create one new genus (*Menderavirus*) including three new species (*Caudovirales*: *Myoviridae*)	2020.101B.
Adriaenssens EM, Tolstoy I, Kropinski AM, Moraru C, Wittmann J	Create one new subfamily (*Migulavirinae*) including two existing genera (*Caudovirales*: *Schitoviridae*)	2020.102B.
Adriaenssens EM, Tolstoy I, Kropinski AM Ramsey J	Create one new genus (*Moabitevirus*) including two new species (*Caudovirales*: *Myoviridae*)	2020.103B.
Adriaenssens EM, Tolstoy I, Kropinski AM	Create one new genus (*Moturavirus*) including one new species (*Caudovirales*: *Myoviridae*)	2020.104B.
Adriaenssens EM, Tolstoy I, Moraru C, Kropinski AM	Create one new genus (*Mufasoctovirus*) including one new species (*Caudovirales*: *Siphoviridae*)	2020.105B.
Adriaenssens EM, Tolstoy I, Kropinski AM, Ramsey J	Create one new genus (*Muldoonvirus*) including two new species (*Caudovirales*: *Myoviridae*)	2020.106B.
Adriaenssens EM, Tolstoy I, Turner D, Kropinski AM	Create one new genus (*Mydovirus*) including six new species in the subfamily *Vequintavirinae* (*Caudovirales*: *Myoviridae*)	2020.107B.
Gonzalez-Serrano R, Dunne M, Rosselli R, Martin-Cuadrado A-B, Grosboillot V, Zinsli LV, Roda-Garcia JJ, Loessner MJ, Rodriguez-Valera F	Create one new genus (*Myoalterovirus*) including one new species (*Caudovirales*: *Myoviridae*)	2020.108B.
Adriaenssens EM, Tolstoy I, Turner D, Kropinski AM	Create one new genus (*Myosmarvirus*) including two new species (*Caudovirales*: *Myoviridae*)	2020.109B.
Adriaenssens EM, Tolstoy I, Turner D, Lueder M, Moineau S, Kropinski AM	Create one new genus (*Nevevirus*) including four new species (*Caudovirales*: *Siphoviridae*)	2020.110B.
Kropinski AM, Tolstoy I, Turner D, Adriaenssens EM	Create one new genus (*Nylescharonvirus*) including two new species (*Caudovirales*: *Myoviridae*)	2020.111B.
Adriaenssens EM, Tolstoy I, Kropinski AM, Moraru C, Wittmann J	Create one new genus (*Pacinivirus*) including two new species (*Caudovirales*: *Schitoviridae*)	2020.112B.
Adriaenssens EM, Tolstoy I, Kropinski AM Ramsey J	Create one new genus (*Parlovirus*) including one new species (*Caudovirales*: *Podoviridae*)	2020.113B.
Roux S, Krupovic M	Create one new family (*Paulinoviridae*) including two genera moved from the family *Inoviridae* (*Tubulavirales*)	2020.114B.
Adriaenssens EM, Tolstoy I, Turner D, Kropinski AM	Abolish one genus (*Pbi1virus*) and rename one species (*Mycobacterium virus Plot*) (*Caudovirales*: *Siphoviridae*)	2020.115B.
Adriaenssens EM, Tolstoy I, Turner D, Kropinski AM	Create one new genus (*Bcepfunavirus*) and create nine new species in the genus *Pbunavirus* (*Caudovirales*: *Myoviridae*)	2020.116B.
van Zyl LJ, Lueder MR, Bishop-Lilly KA, Turner D, Adriaenssens EM, Kropinski AM	Create five new genera and 29 new species in the subfamily *Peduovirinae* (*Caudovirales*: *Myoviridae*)	2020.117B.
Kropinski AM, Adriaenssens EM, Tolstoy I, Barylski J	Create one new genus (*Pimunavirus*) including one new species (*Caudovirales*: *Myoviridae*)	2020.118B.

Adriaenssens EM, Tolstoy I, Turner D, Kropinski AM	Create one new genus (*Plateaulakevirus*) including four new species (*Caudovirales*: *Myoviridae*)	2020.119B.
Adriaenssens EM, Tolstoy I, Kropinski AM, Moraru C, Wittmann J	Create one new genus (*Pokkenvirus*) including one new species (*Caudovirales*: *Schitoviridae*)	2020.120B.
Adriaenssens EM, Tolstoy I, Kropinski AM, Moraru C, Wittmann J	Create one new genus (*Pollockvirus*) including one species moved from the genus *Ithacavirus* (*Caudovirales*: *Podoviridae*)	2020.121B.
Adriaenssens EM, Tolstoy I, Kropinski AM, Jang HB, Sullivan MB, Moraru C, Wittmann J	Create one new subfamily (*Pontosvirinae*) including three new genera (*Caudovirales*: *Schitoviridae*)	2020.122B.
Wittmann J, Adriaenssens EM, Kropinski AM	Create one new genus (*Presleyvirus*) including one new species (*Caudovirales*: *Schitoviridae*)	2020.123B.
Adriaenssens EM, Tolstoy I, Kropinski AM Ramsey J	Create one new genus (*Privateervirus*) including two new species (*Caudovirales*: *Podoviridae*)	2020.124B.
Kropinski AM, Adriaenssens EM, Moraru C	Create one new genus (*Pseudotevenvirus*) including nine species in the subfamily *Tevenvirinae* (*Caudovirales*: *Myoviridae*)	2020.125B.
Adriaenssens EM, Tolstoy I, Kropinski AM, Moraru C, Wittmann J	Create one new genus (*Pylasvirus*) including two new species (*Caudovirales*: *Podoviridae*)	2020.126B.
Adriaenssens EM, Tolstoy I, Turner D, Lueder M, Mahony J, Neve H, Moineau S, Kropinski AM	Create one new genus (*Questintvirus*) including one new species (*Caudovirales*: *Siphoviridae*)	2020.127B.
Ma R, Zhang R	Create one new subfamily (*Queuovirinae*) including one new genus (*Amoyvirus*) and three existing genera (*Caudovirales*: *Siphoviridae*)	2020.128B.
Adriaenssens EM, Tolstoy I, Moraru C, Kropinski AM, Kaliniene L	Rename one genus (*Klausavirus* - formerly *Radnorvirus*) and create three new species (*Caudovirales*: *Myoviridae*)	2020.129B.
Torres-Barceló C, Trotereau A	Create seven new genera and 13 new species of Ralstonia phages (*Caudovirales*)	2020.130B.
Adriaenssens EM, Tolstoy I, Turner D, Holmfeldt K, Šulčius S, Kropinski AM	Create one new genus (*Ravarandavirus*) including three new species (*Caudovirales*: *Siphoviridae*)	2020.131B.
Kropinski AM, Turner D, Adriaenssens EM	Create two new species and move the species *Rhodococcus virus RGL3* to the genus *Rerduovirus* (*Caudovirales*: *Siphoviridae*)	2020.132B.
Adriaenssens EM, Tolstoy I, Kropinski AM, Moraru C, Wittmann J	Create one new subfamily (*Rhodovirinae*) including seven genera (*Caudovirales*: *Schitoviridae*)	2020.133B.
Wittmann J, Adriaenssens EM, Kropinski AM	Create one new genus (*Riverridervirus*) including one new species (*Caudovirales*: *Schitoviridae*)	2020.134B.
Adriaenssens EM, Tolstoy I, Kropinski AM, Łobocka M	Create one new genus (*Rockefellervirus*) including six species (*Caudovirales*: *Siphoviridae*)	2020.135B.
Kropinski AM, Tolstoy I, Adriaenssens EM, Barylski J	Create one new genus (*Rockvillevirus*) including one new species (*Caudovirales*: *Siphoviridae*)	2020.136B.

Adriaenssens EM, Tolstoy I, Turner D, Kropinski AM	Create one new genus (*Ronodorvirus*) including two new species (*Caudovirales*: *Myoviridae*)	2020.137B.
Adriaenssens EM, Tolstoy I, Turner D, Kropinski AM	Create four new species in the genus *Rosemountvirus* (*Caudovirales*: *Myoviridae*)	2020.138B.
Adriaenssens EM, Tolstoy I, Kropinski AM, Moraru C, Wittmann J	Create one new subfamily (*Rothmandenesvirinae*) including four genera (*Caudovirales*: *Schitoviridae*)	2020.139B.
Adriaenssens EM, Tolstoy I, Moraru C, Barylski J, Tong Y, Kropinski AM, Łobocka M	Create one new family (*Rountreeviridae*) including two subfamilies and six genera of predominantly *Staphylococcus* and *Enterococcus* phages (*Caudovirales*)	2020.140B.
Kropinski AM, Tolstoy I, Turner D, Adriaenssens EM	Create one new genus (*Saintgironsvirus*) including one new species (*Caudovirales*: *Myoviridae*)	2020.142B.
Adriaenssens EM, Tolstoy I, Moraru C, Kropinski AM, Barylski J	Create two new genera in the family *Podoviridae* and create one new family (*Salasmaviridae*) including one moved subfamily (*Picovirinae*), two new subfamilies (*Northropvirinae* and *Tatarstanvirinae*), and nine new genera (*Caudovirales*)	2020.143B.
Adriaenssens EM, Tolstoy I, Moraru C, Turner D, Lueder M, Moineau S, Kropinski AM	Create one new genus (*Sandinevirus*) including new one species (*Caudovirales*: *Siphoviridae*)	2020.144B.
Adriaenssens EM, Tolstoy I, Kropinski AM	Create one new genus (*Sarumanvirus*) including two new species (*Caudovirales*: *Myoviridae*)	2020.145B.
Adriaenssens EM, Tolstoy I, Kropinski AM, Moraru C, Wittmann J	Create one new family (*Schitoviridae*) including eight existing subfamilies and 40 existing genera (*Caudovirales*: *Schitoviridae*)	2020.146B.
Kropinski AM, Adriaenssens EM	Create one new genus (*Sendosyvirus*) including two species (*Caudovirales*: *Podoviridae*)	2020.147B.
Lehman SM, Petit MA, Lossouarn J, Tong Y, Tolstoy I, Adriaenssens E	Create ten new species in the subfamily *Sepvirinae* (*Caudovirales*: *Podoviridae*)	2020.148B.
Kropinski AM, Turner D, Adriaenssens EM	Create one new genus (*Shandongvirus*) including one new species (*Caudovirales*: *Myoviridae*)	2020.149B.
Adriaenssens EM, Tolstoy I, Kropinski AM	Create one new genus (*Shirahamavirus*) including one new species (*Caudovirales*: *Myoviridae*)	2020.150B.
Kropinski AM, Tolstoy I, Adriaenssens EM, Barylski J, Piligrimova EG, Kazantseva OA, Nikulin NA, Shadrin AM	Create one new subfamily (*Skryabinvirinae*) including two new genera, each with one new species (*Caudovirales*: *Siphoviridae*)	2020.152B.
Adriaenssens EM, Tolstoy I, Moraru C, Turner D, Lueder M, Moineau S, Kropinski AM	Create 80 new species in the genus *Skunavirus* (*Caudovirales*: *Siphoviridae*)	2020.153B.
Adriaenssens EM, Tolstoy I, Turner D, Kropinski AM	Create one new genus (*Sleepyheadvirus*) including one new species (*Caudovirales*: *Siphoviridae*)	2020.154B.
Kropinski AM, Tolstoy I, Turner D, Adriaenssens EM	Create one new genus (*Sozzivirus*) including three new species (*Caudovirales*: *Siphoviridae*)	2020.155B.

Adriaenssens EM, Tolstoy I, Turner D, Kropinski AM	Create one new genus (*Sparkyvirus*) including one new species (*Caudovirales*: *Siphoviridae*)	2020.156B.
Kropinski AM, Tolstoy I, Adriaenssens EM, Barylski J	Create one new genus (*Spizizenvirus*) including one new species (*Caudovirales*: *Siphoviridae*)	2020.157B.
Adriaenssens EM, Tolstoy I, Turner D, Kropinski AM	Create one new genus (*Squirtyvirus*) including one new species (*Caudovirales*: *Siphoviridae*)	2020.158B.
Adriaenssens EM, Tolstoy I, Turner D, Kropinski AM	Create four new species in the genus *Samwavirus* (*Caudovirales*: *Siphoviridae*)	2020.159B.
Kropinski AM, Adriaenssens EM, Barylski J	Create one new genus (*Takahashivirus*) including one existing species (*Bacillus virus PBS1*) (*Caudovirales*: *Myoviridae*)	2020.160B.
Adriaenssens EM, Tolstoy I, Kropinski AM Barylski J	Create one new genus (*Tandoganvirus*) including one new species (*Caudovirales*: *Siphoviridae*)	2020.161B.
Adriaenssens EM, Tolstoy I, Moraru C, Kropinski AM	Create 53 new species, rename one species and move one species in the genus *Tequatrovirus* (*Caudovirales*: *Myoviridae*)	2020.162B.
Adriaenssens EM, Tolstoy I, Moraru C, Turner D, Lueder M, Neve H, Mahony J, Moineau S, Kropinski AM	Create one new genus (*Teubervirus*) including five new species (*Caudovirales*: *Siphoviridae*)	2020.163B.
Adriaenssens EM, Tolstoy I, Turner D, Kropinski AM	Create one new genus (*Thetabobvirus*) including three new species (*Caudovirales*: *Siphoviridae*)	2020.164B.
Adriaenssens EM, Tolstoy I, Moraru C, Kropinski AM	Create one new subfamily (*Trabyvirinae*) including two new genera (*Caudovirales*: *Siphoviridae*)	2020.165B.
Adriaenssens EM, Tolstoy I, Kropinski AM, Łobocka M	Create 17 new species in the genus *Triavirus* (*Caudovirales*: *Siphoviridae*)	2020.166B.
Adriaenssens EM, Tolstoy I, Moraru C, Kropinski AM	Create one new genus (*Triplejayvirus*) including one new species (*Caudovirales*: *Siphoviridae*)	2020.167B.
Adriaenssens EM, Tolstoy I, Moraru C, Kropinski AM	Create four new species and abolish one species in the genus *Tunavirus* (*Caudovirales*: *Drexlerviridae*)	2020.169B.
Adriaenssens EM, Tolstoy I, Kropinski AM Oliveira H, Turner D, Moraru C	Create one new subfamily (*Twarogvirinae*) including five new genera (*Caudovirales*: *Podoviridae*)	2020.170B.
Adriaenssens EM, Tolstoy I, Turner D, Lueder M, Nakai T, Kropinski AM	Create one new genus (*Uwajimavirus*) including one new species (*Caudovirales*: *Siphoviridae*)	2020.171B.
Adriaenssens EM, Tolstoy I, Turner D, Lueder M, Moineau S, Mahony J, Kropinski AM	Create one new genus (*Vedamuthuvirus*) including five new species (*Caudovirales*: *Siphoviridae*)	2020.172B.
Adriaenssens EM, Tolstoy I, Moraru C, Kropinski AM	Create one new genus (*Veterinaerplatzvirus*) including one new species (*Caudovirales*: *Drexlerviridae*)	2020.173B.
Adriaenssens EM, Tolstoy I, Moraru C, Kropinski AM	Create one new genus (*Vibakivirus*) including one new species (*Caudovirales*: *Myoviridae*)	2020.174B.

Wittmann J, Adriaenssens EM, Kropinski AM	Create one new genus (*Waedenswilvirus*) including one new species (*Caudovirales*: *Schitoviridae*)	2020.175B.
Adriaenssens EM, Tolstoy I, Kropinski AM, Turner D, Łobocka M	Create one new genus (*Warsawvirus*) including one new species (*Caudovirales*: *Autographiviridae*)	2020.176B.
Adriaenssens EM, Tolstoy I, Moraru C, Kropinski AM	Create eight new species and abolish one species in the genus *Warwickvirus* (*Caudovirales*: *Drexlerviridae*)	2020.177B.
Kropinski AM, Tolstoy I, Adriaenssens EM, Barylski J	Create one new genus (*Waukeshavirus*) including two new species (*Caudovirales*: *Siphoviridae*)	2020.178B.
Adriaenssens EM, Tolstoy I, Moraru C, Kropinski AM	Create 16 new species and abolish two species in the genus *Webervirus* (*Caudovirales*: *Drexlerviridae*)	2020.179B.
Adriaenssens EM, Tolstoy I, Turner D, Kropinski AM	Create one new genus (*Whackvirus*) including one new species (*Caudovirales*: *Siphoviridae*)	2020.180B.
Adriaenssens EM, Tolstoy I, Mahony J, Neve H, Moineau S, Kropinski AM	Create one new genus (*Whiteheadvirus*) including one new species (*Caudovirales*: *Siphoviridae*)	2020.181B.
Kropinski AM, Tolstoy I, Adriaenssens EM, Turner D	Create one new genus (*Wumpquatrovirus*) including one new species (*Caudovirales*: *Podoviridae*)	2020.182B.
Kropinski AM, Tolstoy I, Adriaenssens EM, Turner D	Create one new genus (*Wumptrevirus*) including two species (*Caudovirales*: *Podoviridae*)	2020.183B.
Adriaenssens EM, Tolstoy I, Kropinski AM, Moraru C	Create one new genus (*Yonseivirus*) including three new species (*Caudovirales*: *Siphoviridae*)	2020.184B.
Wittmann J, Adriaenssens EM, Kropinski AM	Create one new genus (*Zicotriavirus*) including two new species (*Caudovirales*: *Schitoviridae*)	2020.185B.
Bischoff V, Adriaenssens EM, Kropinski AM, Duhaime M, Moraru C	Create one new family (*Zobellviridae*) including one new subfamily (*Cobavirinae*), seven new genera and 12 new species (*Caudovirales*)	2020.187B.
Adriaenssens EM, Tolstoy I, Kropinski AM, Moraru C, Wittmann J	Create one new genus (*Zurivirus*) including one new species (*Caudovirales*: *Schitoviridae*)	2020.188B.
